# The reliability of knee joint position testing using electrogoniometry

**DOI:** 10.1186/1471-2474-9-6

**Published:** 2008-01-22

**Authors:** Pagamas Piriyaprasarth, Meg E Morris, Adele Winter, Andrea E Bialocerkowski

**Affiliations:** 1 School of Physiotherapy, The University of Melbourne, Victoria, Australia; 2 Department of Physiotherapy, Caulfield General Medical Centre, Victoria, Australia

## Abstract

**Background:**

The current investigation examined the inter- and intra-tester reliability of knee joint angle measurements using a flexible Penny and Giles Biometric^® ^electrogoniometer. The clinical utility of electrogoniometry was also addressed.

**Methods:**

The first study examined the inter- and intra-tester reliability of measurements of knee joint angles in supine, sitting and standing in 35 healthy adults. The second study evaluated inter-tester and intra-tester reliability of knee joint angle measurements in standing and after walking 10 metres in 20 healthy adults, using an enhanced measurement protocol with a more detailed electrogoniometer attachment procedure. Both inter-tester reliability studies involved two testers.

**Results:**

In the first study, inter-tester reliability (ICC_[2,10]_) ranged from 0.58–0.71 in supine, 0.68–0.79 in sitting and 0.57–0.80 in standing. The standard error of measurement between testers was less than 3.55° and the limits of agreement ranged from -12.51° to 12.21°. Reliability coefficients for intra-tester reliability (ICC_[3,10]_) ranged from 0.75–0.76 in supine, 0.86–0.87 in sitting and 0.87–0.88 in standing. The standard error of measurement for repeated measures by the same tester was less than 1.7° and the limits of agreement ranged from -8.13° to 7.90°. The second study showed that using a more detailed electrogoniometer attachment protocol reduced the error of measurement between testers to 0.5°.

**Conclusion:**

Using a standardised protocol, reliable measures of knee joint angles can be gained in standing, supine and sitting by using a flexible goniometer.

## Background

This study evaluated the inter-tester reliability and intra-tester reliability of electrogoniometric measures of sagittal knee positions in supine, sitting and standing. Knee joint angle measurements are performed as a part of joint assessments and to evaluate treatment outcomes in elite athletes [1,2] and patients with medical conditions such as arthritis [3,4] and stroke [5]. Standard handheld goniometers are often used in clinical settings to quantify static knee joint positions. Measurement of knee joint angles relies upon the accurate identification of the centre of rotation of the knee [6]. Because the centre of knee joint rotation changes with movement [7,8], it can be difficult to track using a hand held goniometer. Three dimensional motion analysis systems accurately locate the centre of knee joint rotation yet are expensive, time consuming to use and require the skills of well trained users [9,10]. Alternatively, the relative positions of the thigh and leg can be measured using electrogoniometry [11,12], gravity-based goniometers [13] or fluid-based inclinometers [14].

Single axis and triaxial electrogoniometers enable quick measurement of joint positions and continuous knee joint motion [11]. A potential source of error is misalignment of the electrogoniometer to the anatomical axis of the knee joint, leading to difficulties in determining the zero position [15]. Another source of error can be electrogoniometer slippage during movement [15]. Flexible light weight electrogoniometers have recently been developed that enable the capture of movements in all planes [16]. Being lighter than traditional electrogoniometers, they do not have the same propensity to slip. Moreover, they do not require the tester to locate the centre of rotation of the knee joint because the relative position of the thigh to the leg determines the knee joint angle.

Although electrogoniometers have been used for measurements of knee joint angle in a number of studies, few have reported their reliability in the sagittal plane [17-19]. One study in healthy people reported good intra-tester reliability for a triaxial electrogoniometer for the measurement of knee joint motion during walking [17]. No studies have reported the inter-tester and intra-tester reliability of electrogoniometry for static knee joint measurements in different testing positions. This is despite the use of electrogoniometers for measurements in sitting and standing [18,20]. How much slippage of the electrogoniometer attachment occurs during dynamic tasks such as walking has not been reported. Moreover, whether electrogoniometers should be left on the leg during repeated testing or taken off and reattached remains unclear. These factors could generate measurement error from slippage on the skin or inconsistent repositioning of the electrogoniometer end blocks. Therefore, two studies were conducted to investigate reliability and measurement error. The first investigated both inter-tester and intra-tester reliability of knee joint measurements in supine, sitting and standing using a flexible electrogoniometer. Based on the results of this study, a more detailed testing protocol was devised to minimise measurement error arising from electrogoniometer re-attachment. The second study evaluated the inter- and intra-tester reliability of the detailed protocol and the effects of walking on subsequent measurements.

## Methods

### Instrumentation

**Figure 1:**
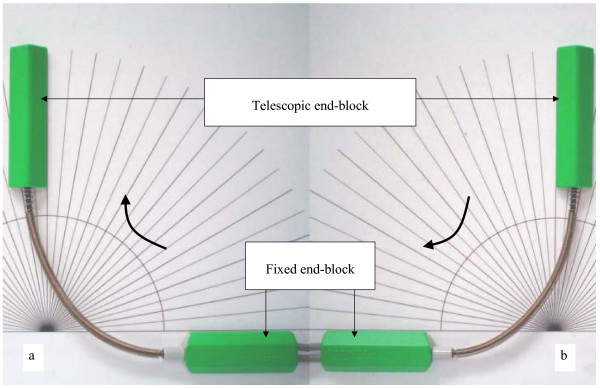
Validation of the electrogoniometer: (a) positive values, (b) negative values.

A Penny and Giles Biometrics^® ^(P & GB) twin axis electrogoniometer (SG 150) (Cwmfelinfach, UK) was used to quantify knee joint angles and knee motion. The electrogoniometer is comprised of optical fibres to measure motion, a fixed end-block and a telescopic end-block (Figure 1). The mechanical signals from the measuring element in the end-blocks were converted into a digital signal by a datalog acquisition unit which connected the electrogoniometer to a display unit. Because a frequency rate of approximately 200 Hz was previously used for measuring knee joint movement in functional activities [17,21], we also selected a sampling rate of 200 Hz.

Prior to the reliability studies, we examined the accuracy of the electrogoniometer against a laboratory perspex template with engraved increments of 5° (Figure 1). By moving the telescopic end-block clockwise towards the fixed end-block, joint angles were recorded as positive values. By moving the telescopic end-block anticlockwise to the fixed end-block, negative values of angles were recorded. The fixed end-block was adhered to the template at a known position of 0° using double adhesive tape. The telescopic end-block was then moved to a desired angle. Calibrations were performed every five degrees within the range of 0°–180° in random order and each angle was measured 10 times to establish consistency of measurement. The angle reading outputs of the electrogoniometer in both directions were calibrated using this procedure. The differences between electrogoniometer angles and the reference angles were recorded. Using this validation procedure, the electrogoniometer was shown to have a measurement error of 0.04°.

An adjustable plinth with three separate sections (Metron Medical Australia, Edwardstown) was used for standardisation of knee joint positions at three angles from the horizontal plane (0°, 45°, 75°) in sitting and supine. These angles were chosen to represent knee joint positions in flexion and extension. A flat laminated paper with three interval lines was used to standardise placement of the participant's heels in standing. A vertical wooden board was used to set the position of the knee joint in either flexion or extension.

### Study One

#### Participants

Thirty five unimpaired volunteers participated in the inter-tester reliability study. They were recruited as a sample of convenience from staff, undergraduate and postgraduate students at La Trobe University. The mean age of participants was 31 years (19–44 years). There were 26 women and nine men. Of the 35 participants, 20 had left knees tested and 15 had right knees tested. Twenty-two participants from the inter-tester reliability study were also measured to determine intra-tester reliability. Fifteen additional participants were recruited to establish intra-tester reliability. Therefore for the intra-tester reliability study, 37 unimpaired volunteers ranging from 19–45 years (mean 31 years) participated. There were 23 women and 14 men with 19 left knees and 18 right knees tested. To be included, participants needed to be healthy adults who had a full range of motion of the knee joint and no history of knee injury. A blocked random numbers table was computer generated. The leg being tested was assigned from this random numbers table. Written consent was obtained from all participants. This study was approved by the Faculty of Health Sciences Ethics Committee, La Trobe University, Australia (FHEC 05/35).

A sample size of at least 35 participants was used, based on Cohen' s formula [22] with alpha set at 0.05 and 80% power. For two assessors at least 35 participants were required to yield 80% power for alpha at 0.8 [23].

#### Testers

Two testers (PP and PF) who had 1–3 months experience using the electrogoniometer participated in the inter-tester reliability study. One tester (PP) participated in the intra-tester reliability study. Both testers were physiotherapists with more than three years clinical experience involving the use of standard goniometers.

#### Procedure

Participants were asked to wear shorts for ease of attachment of the electrogoniometer to the lateral side of the knee joint. Shoes and socks were removed during testing to accurately locate the lateral malleolus. The tested knee was wrapped with thin foam to minimise any visible skin markings made by the electrogoniometer which may have influenced the testers.

For the measurements in standing, each tester attached the electrogoniometer to the knee joint in the neutral knee position, in accordance with manufacturer's guidelines [24]. The neutral knee position was defined as a relationship between the thigh and the leg in the anatomical position [25]. The telescopic block was placed in parallel to an imaginary line between the head of the fibula and the lateral malleolus. The fixed end-block was placed in parallel to an imaginary line between the greater trochanter and the lateral condyle of the femur. In the neutral knee position, the electrogoniometer was set at zero degrees and this was confirmed with a hand held goniometer. To prevent slippage during knee joint motion, the end-blocks were adhered to the test leg with double sided adhesive tape and further secured in place with adhesive tape. Electrogoniometer readings recorded knee joint angular displacements relative to zero.

**Figure 2:**
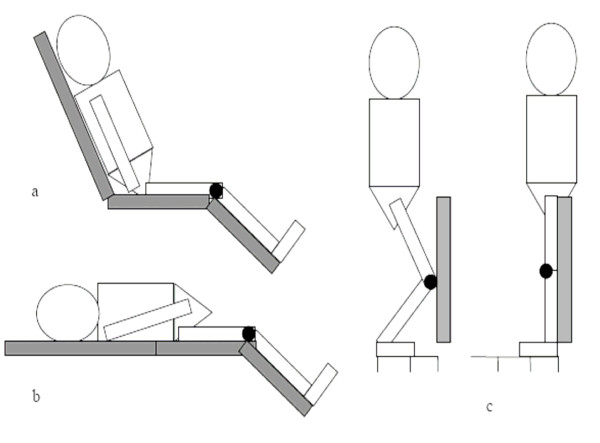
Electrogoniometry in sitting (a), supine (b) and standing (c).

The knee joint was measured in three different testing positions – neutral and two flexion positions (designated knee flexion position 1 and knee flexion position 2). For measuring the neutral knee position, participants were asked to stand facing away from the wall at a distance where their calves remained touching the vertical wooden board. They were instructed to straighten their knee from a flexed position to lightly touch the calf against the board. For measurements of knee joint flexion, each participant stood facing a vertical wooden board. Two standardised heel placements with distances of 13.5 centimetres and 22.5 centimetres from the board enabled measurement of two different knee flexion angles (Figure 2). These were average distances obtained from our pilot study on people with different heights, who could perform the task without compensation at the hip joint or the trunk. Participants were asked to bend their knee so as to lightly touch the board in front of the knee, while maintaining contact of their heels on the ground. The participants were also asked to keep their back straight to minimise variations in trunk position. Knee joint angles were recorded in this position. Knee positions were dependent on the position of heel placement and where the knee touched the vertical wooden board. Therefore, knee flexion angles varied among individuals according to their leg length. Ten measurements were performed for each knee position to quantify the consistency of repeated electrogoniometric measurements. After measurements were taken in standing, the electrogoniometer was removed.

The electrogoniometer was re-attached to the knee in the supine position for the measurements in supine and sitting. The participant's knee joint line was positioned directly over the joint between the middle and end sections of the plinth. In the neutral knee position, the electrogoniometer was attached to the lateral side of knee joint using the same attachment protocol as for standing. In sitting, the proximal end of the plinth was set upwards at approximately 75° to support the participant's back (Figure 2). The remaining procedures were the same as measurements in supine.

The knee joint was measured at three different positions in supine and sitting. The neutral knee position and two knee flexion positions, at plinth angles of 45° and 75°, were quantified (designated knee flexion position 1 and knee flexion position 2, respectively). These angles were selected to enable standardisation of knee joint positions and across 10 repetitions.

Each participant was tested in all three positions. Testers used the same protocol for electrogoniometer attachment and the same order of testing. The first tester always removed the electrogoniometer from the participant's leg after completing the measurements to ensure independence of observations. Participants were given a short break before the second tester reattached the electrogoniometer on the same leg. To minimise series effects, a computerised random numbers table was generated, defining the order of testers, leg tested, testing position and knee joint position.

The intra-tester reliability phase of the study was conducted by one tester (PP) measuring knee joint angles on two occasions on the same participants on the same day. The procedure for preparing the participant knee joint measurements was used as described above with an identical order for the first and the second measurements.

### Statistical analysis

All data for knee joint angle measurements were recorded using Biometrics^® ^analysis software and further analyses were conducted using SPSS 11.5 software for Windows. For the reliability analyses, the means and standard deviations of 10 measurements in each knee position were used. Intraclass Correlation Coefficients (ICC_[2,10]_) were used to estimate the inter-tester reliability and ICCs_[3,10] _were used to estimate the intra-tester reliability [26]. Measurement error was estimated using the standard error of measurement (SEM) [23,27]. The 95% CI of the mean difference of electrogoniometer averaged measurements were determined using limits of agreement for both measurements between testers and within the same tester [28]. The upper and the lower limits of agreements were computed from two standard deviations of the mean difference.

It was predicted that little variation would occur across measurement trials for the neutral knee position. Therefore ICCs were considered to be inappropriate to use because they cannot be computed when the variance is close to zero [29]. For the measurement of the neutral knee position, only the SEM was calculated.

## Results

### Study One

#### Inter-tester reliability

The ICCs_[2,10] _for inter-tester reliability of knee joint measurements ranged from 0.57 to 0.80 across three testing positions with the error of measurement between testers ranging from 1.48° to 3.55° (Table 1). Limits of agreement for measurements of the three knee joint positions ranged from -7° to 14° in sitting, -9.7° to 12.2° in supine and -12.5° to 7.2° in standing (Table 2). The position of the knee joint affected the error of measurement with larger angles tending to have the greater error of measurement. The mean difference of the 10 measurements by two testers was less than 1.25° and the limits of agreement ranged from -6.7° to 8.3°. This was with the exception of measures of knee flexion (position 2) in standing, sitting and supine and knee flexion (position 1) in standing. For these knee positions, the mean difference was greater, ranging from -3.9° to 3.5°.

**Table 1:** Inter-tester reliability and intra-tester reliability of knee joint angle measurements in sitting and supine

Position	Inter-tester reliability (n = 35)		Intra-tester reliability (n = 37)	
	
	ICC_[2,10]_	SEM (°)	ICC_[3,10]_	SEM (°)
Sitting				
Neutral knee position	-	2.31	-	1.21
Knee flexion position 1	0.79	1.48	0.87	0.90
Knee flexion position 2	0.68	2.97	0.86	1.25
Supine				
Neutral knee position	-	1.97	-	0.80
Knee flexion position 1	0.71	2.03	0.75	1.51
Knee flexion position 2	0.58	3.55	0.76	1.74
Standing				
Neutral knee position	-	1.61	-	1.61
Knee flexion position 1	0.57	3.20	0.87	1.37
Knee flexion position 2	0.80	1.94	0.88	1.31

ICC = Intraclass correlation coefficient, SEM = standard error of measurement

See the details of knee positions in text.

**Table 2:** Means, standard deviation and limits of agreement for the inter-tester reliability of knee joint angle measurement

Measures	Mean1(SD) (°)	Mean2(SD) (°)	Mean Diff(SD) (°)	Lower limit (°)	Upper limit (°)
Sitting					
Neutral knee	4.86(3.02)	4.41(2.46)	0.45(3.21)	-5.97	6.87
Knee flexion 1	20.89(3.95)	19.94(3.87)	0.95(3.23)	-5.51	7.41
Knee flexion 2	36.34(5.72)	32.87(4.94)	3.48(5.24)	-7.00	13.96
Supine					
Neutral knee	2.55(1.89)	2.74(1.65)	-0.18(2.30)	-4.78	4.42
Knee flexion 1	18.91(4.05)	18.13(3.88)	0.78(3.76)	-6.74	8.30
Knee flexion 2	29.69(4.52)	28.44(5.51)	1.25(5.48)	-9.71	12.21
Standing					
Neutral knee	2.91(2.20)	2.59(2.03)	0.32(2.38)	-4.44	5.08
Knee flexion 1	9.33(4.90)	11.87(3.98)	-2.54(4.88)	-12.30	7.22
Knee flexion 2	24.45(5.49)	28.31(5.16)	-3.85(4.33)	-12.51	4.18

Mean1 = Mean of the measurements by the first tester, Mean2 = mean of the measurement by the second tester, SD = standard deviation Mean Diff = mean difference

#### Intra-tester reliability

The intra-tester reliability (ICC_[3,10]_) of knee joint angle measurements for the three testing positions ranged from 0.75–0.88. The standard error of measurement for repeated measurements by the same tester ranged from 0.8° to 1.7° (Table 1). Less error of measurement was found in more flexed positions in sitting and standing, and less error was found in the neutral knee position in supine. In sitting and standing, the error of measurement was greater in the neutral position than for the flexed positions. The mean difference for the average 10 measurements between the first and the second sessions was less than 1.0°. Limits of agreement for intra-tester reliability ranged from -8.1° to 7.9° for the measurements in all positions (Table 3).

**Table 3:** Means, standard deviation and limits of agreement for the intra-tester reliability of knee joint angle measurement

Measures	Mean1(SD) (°)	Mean2(SD) (°)	Mean Diff(SD) (°)	Lower limit (°)	Upper limit (°)
Sitting					
Neutral knee	3.41(2.17)	3.54(1.99)	-0.12(2.09)	-4.30	4.06
Knee flexion 1	18.28(3.69)	18.64(3.66)	-0.35(2.50)	-5.35	4.65
Knee flexion 2	30.83(4.83)	31.11(4.85)	-0.27(3.37)	-7.01	6.47
Supine					
Neutral knee	2.24(1.68)	2.17(1.57)	0.07(1.51)	-2.95	3.09
Knee flexion 1	16.83(3.77)	16.94(3.08)	-0.11(3.06)	-6.23	6.01
Knee flexion 2	27.18(4.07)	28.15(4.23)	-0.97(3.58)	-8.13	6.19
Standing					
Neutral knee	3.44(2.78)	2.93(2.57)	0.5(2.72)	-4.94	5.94
Knee flexion 1	12.16(5.85)	12.41(4.98)	-0.25(3.74)	-7.73	7.23
Knee flexion 2	27.60(6.35)	27.42(5.63)	0.18(3.86)	-7.54	7.90

Mean1 = Mean of the first measurement, Mean2 = mean of the second measurement, Mean Diff = mean difference, SD = standard deviation

### Study Two

For the first study, measurement errors in supine and sitting came from measurement technique rather than the attachment procedure. The error of measurement for the neutral knee position in standing appeared to be related to electrogoniometer attachment protocol and variable knee positioning across the 10 movement repetitions (SEM = 1.6°). Differing amounts of calf pressure on the board may also have contributed to the error of measurement. Because it is important to obtain reliable measurements in the neutral knee position, the second reliability study was conducted. This used a more detailed measurement protocol in the standing position. In addition, this study investigated the effect of walking on subsequent measurements and the error associated with electrogoniometer reattachment.

#### Participants

Twenty new unimpaired volunteers were recruited as a sample of convenience from physiotherapy students of the School of Physiotherapy at The University of Melbourne, Australia. The mean age for the participants in this group was 20.1 years (18–30 years). There were 19 females and one male. All participants had their right knees tested as it was found from the first study that no differences existed between the left knee and right knees. The inclusion criteria were the same as for the first study. This study was approved by the Human Research Ethics Committee, The University of Melbourne. All participants provided written consent (0710578.1).

#### Testers

Two testers (PP and EG) were involved in this study. EG received a two hour training session in attaching and operating the P & GB electrogoniometer. The tester PP had experience in using the electrogoniometer as described in the first study.

#### Procedure

Barefoot standing with toes on a straight line on the floor was used to standardise the placement of the feet. Each tester attached the electrogoniometer to the right knee joint in the neutral knee position in standing. A standardised procedure of attachment was used. The end-blocks were directly adhered to the participant's legs with double sided adhesive tape and further secured in place with non allergic adhesive tape. The zero position was confirmed with a hand held goniometer.

Knee joint angles in neutral were recorded while the participants were standing still for one minute and after walking 10 metres. The electrogoniometer was reset to the zero degrees before each of the measurements and calibrated against a hand held goniometer. The electrogoniometer was then removed and the participants had a one minute rest. After resting, the electrogoniometer was reattached to the lateral side of the knee, in standing. This standardised protocol was performed by each tester within the same session. A one minute break was provided between tests by the same tester and a further one minute before the second tester reattached the electrogoniometer. This was minimised visible skin markings from the electrogoniometer which might have influenced the testers. The order of testers was randomised. Half of the participants had PP as the first tester and another half had EG as the first tester.

### Statistical analysis

As with Study One, it was predicted in this study that little variation would occur across measurement trials. Therefore, ICCs were considered to be inappropriate to use because they cannot be computed when the variance is close to or is zero [29]. Only the SEM was calculated in this study to estimate measurement error. Means and standard deviations were used to calculate the SEM. Limits of agreement [28] were also used to determine the association of electrogoniometer measurements between testers and for the repeated measurements by the same tester.

### Results – Study Two

For inter-tester reliability, the standard error of electrogoniometric measurement in standing, after walking and after the reattachment between testers, ranged from 0.5° to 3.3° (Table 4). Maximum variation of 1.0° was found in only 20% of the measurements which represented four participants in static standing. Limits of agreement for the means and the standard deviation of the knee joint angle measurements measured by two testers ranged from -6.0° to 4.9° with a mean difference less than 0.6°. For intra-tester reliability, the standard error of measurement after reattachment by individual testers, ranged from 1.3° to 2.3° (Table 4). Mean differences for individual testers for reattachment ranged from -1.5° to -1.9° (Limits of agreement ranged from -6.7° to 2.9°).

**Table 4:** Inter-tester reliability and intra-tester reliability for measurements in Study Two

Measures	Mean1 (SD) (°)	Mean2 (SD) (°)	Mean Diff (°)	SEM (°)	Lower limit (°)	Upper limit (°)
Inter-tester						
One minute standing	0.2(0.41)	0.2(0.41)	0.00(0.56)	0.54	-1.1	1.1
After walking	1.8(1.44)	2.35(1.66)	-0.55(2.70)	3.33	-5.95	4.85
Reattachment	1.5(1.40)	1.9(2.40)	-0.40(2.62)	2.45	-5.64	4.84
Intra-tester						
Reattachment						
Tester 1	0.0	1.5(1.40)	-1.50(1.40)	1.30	-4.30	1.30
Tester 2	0.0	1.9(2.40)	-1.90(2.40)	2.25	-6.70	2.90

Mean Diff = mean difference, SD = standard deviation, SEM = standard error of measurement, Mean1 = Mean of the first tester or by the first measurement, Mean2 = mean of the second tester or by the second measurement

## Discussion

Although electrogoniometers have been used in a number of studies to quantify knee joint angles [17-19,30], inter-tester and intra-tester reliability have not been adequately examined. This study quantified knee joint angles in three starting positions (supine, sitting, standing) and following walking. The results showed that using a standardised protocol minimises measurement error, enabling reliable measures of performance.

Measurement error can originate from the electrogoniometer itself, the user and the participant [31]. The measurement error from electrogoniometry (0.8°–3.6°) in the current study was comparable to previous reports (3.0°) [16,17]. Whereas our validity study used a perspex template to confirm angle readings, Rowe et al [16] and Isacson et al [17] validated electrogoniometery using humans, obtaining similar results. The similar ranges of measurement error suggest that electrogoniometers can be used for both static and dynamic knee joint angle measurements.

Intra-tester reliability was higher than inter-tester reliability for all testing positions. This agrees with previous reports on goniometric measurement of the knee joint [6,13,32]. In addition, one electrogoniometry study has shown that measurements performed by the same tester were highly repeatable [17]. It is likely that the same tester is able to re-attach the electrogoniometer in the same position more consistently and accurately than different testers.

Different testing positions influenced reliability, with the sitting position being more reliable both within and between testers. This was confirmed by greater ICCs and lower SEMs for both inter-tester and intra-tester reliability in sitting compared to the supine position. Larger ICCs indicate greater association between measurements, while lower SEMs reflect less variation within subjects [29,31]. Notwithstanding, the difference in measurement error between the sitting and supine positions was less than 0.6°. Measurement error in standing was not compared with supine or sitting because different knee positions were measured for the standing trials. Direct comparisons of ICC values with previous studies could not be made because no ICC data have previously published.

In the current study measurements were obtained for two different flexion angles in sitting, supine and standing. These were found to be moderately reliable when tested by different testers. There was good to excellent intra-tester reliability. The electrogoniometer was reliable for the measurement of knee joint flexion angles at least within the range of 0–90°.

Measurements in sitting and supine that required alignment of the knee joint line to the plinth were more likely to generate measurement error because of visual alignment discrepancies. To improve the reliability of the measurement procedure in sitting and supine, a bony landmark, rather than the knee joint line, could be used as a reference point. In standing, error could have arisen from variable knee positions across the 10 repetitions as a consequence of different amounts of calf pressure on the board. Therefore, careful re-positioning of the participant's knee is needed when several measurements are taken.

Accurate electrogoniometer attachment is important in obtaining valid measurements of knee positions. Identifying the zero position requires placing the two end-blocks of the electrogoniometer parallel with each other [16,17]. Different limb contours could influence the alignment of the goniometer. The results from the second study showed that using a more detailed electrogoniometer attachment protocol reduced the measurement error. The use of the thin foam is not recommended as it increased slippage, despite preventing visual marks.

The limitations of this investigation included the use of a plinth of fixed length to position the knee and leg. Not all participants could place their heel on the plinth and this may have contributed to measurement error. Data were collected following walking, however electrogoniometers could also be used to measure knee joint angles during walking or other dynamic activities. In addition, only three knee angles were tested, and all were less than 90°. It may not be appropriate to predict the error of knee joint measurement for angles greater than 90°. Based on our results, it is predicted that measurement of greater knee flexion angles would be less accurate than for 90°.

In terms of clinical utility, electrogoniometry was found to be highly accurate and highly sensitive for detecting changes in knee joint angles over time. This result implies that changes greater than 3.55° can be considered to be a clinical significant regardless of the testing position used. To minimise error, measurements can be performed in sitting rather than supine. Difficulty in aligning the knee joint line to the plinth in supine resulted in large measurement errors. In addition, measurements in supine are not as functional as for sitting.

The current study examined the measurement error introduced by the reattachment of the electrogoniometer as well as the error from different testers. Measurement error from the reattachment of the electrogoniometer using the standardised procedure in standing was less than 2.5°. If possible, the electrogoniometer should be left attached to the leg between tests because reattachment is another source of measurement error. If it is necessary to remove the electrogoniometer for treatment, a mark indicating electrogoniometer position should be placed on the leg as a reference for subsequent electrogoniometer attachments and measurements. One tester should also perform all measurements as measurement error is less compared with two testers.

In the current study, 10 movement repetitions were used. It is acknowledged that 10 repetitions may not always be possible due to changes in knee joint stiffness, pain or time constraints. In terms of generalisability, the reliability of a single measurement (the first measurement) in subsequent analyses was found to be less reliable in all testing positions compared with the reliability gained from the average of 10 measurements.

Although reliability depends on the population tested, the knee joint angle measurement protocol can be applied to individuals with different leg contours. However, the electrogoniometer must be aligned, so that the two end-blocks are parallel and in the same plane.

## Conclusion

Flexible, light weight electrogoniometers are reliable for measuring static knee joint angles in supine, sitting and standing. Using a standardised measurement protocol, the error of measurement was found to be less than 3.5° between different testers and less than 1.7° when repeated measurements were repeated by the same tester. Measurement error could be minimised using a standard attachment protocol and standardised measurement procedures.

## Competing interests

The author(s) declare that they have no competing interests.

## Authors' contributions

PP participated in the design of the study, data collection, analysis and drafting the manuscript. MEM participated in the design and analysis of the study and commenting on the manuscript. AW and AEB participated in data analysis and interpretation and commenting on drafts of the manuscript. All authors read and approved the final manuscript.

## Pre-publication history

The pre-publication history for this paper can be accessed here:


